# Glances at the Hospitals

**Published:** 1900-06-16

**Authors:** 


### GLANCES AT THE HOSPITALS.

### THE PRINCESS ALICE MEMORIAL HOSPITAL, EASTBOURNE.

This, the sixteenth annual report, shows that 379 cases were treated in the hospital during the year 1899, being an increase of 36 on the number for 1898. On January 1st the hospital contained 25 patients; 354 were admitted; 267 were discharged cured; 41 relieved; 3 not improved; 4 left at their own request; 17 died; and 22 remained in the hospital on December 31st. No proper medical statistics are incorporated with the report, but we learn from the managers' statement that there were 56 accidents admitted, and 25 major operations were performed. The average duration of each patient's residence was 25½ days, being only a few hours longer than the average for 1898; but the average number of patients resident rose from 22 to 26. The average cost of living for patients and staff was one shilling per head per day, which must be looked on as a satisfactory maintenance rate for a hospital of this size. The income amounted to £2,044, and the expenditure to £2,134. The annual subscriptions and the Hospital Sunday offertories show a considerable increase, while the Hospital Saturday Fund and the donations have both decreased. A new wing is being added to the hospital at an expense of £3,500, and the furnishing will amount to £500 extra. Of this sum £1,734 have been subscribed. The building is almost finished, and there will be a debt of at least £2,000 to meet in one way or another, or a heavy annual item for interest to pay. Such debts always cripple the usefulness of any institution, and we doubt whether it is advisable to begin building operations until the sum required to be spent is actually in the managers' hands. The matron is Miss Ramsay.

### THE RADCLIFFE INFIRMARY, OXFORD.

During 1899 there were treated in this hospital 1,872 in-patients, being an increase of 348 on the previous year. The out-patients were 3,130, being an increase of 111 on the number for 1898. These numbers show a total of upwards of 5,000 cases; but in addition to those we find there were 3,718 cases of minor accidents, casual applicants, and dental cases. The daily average resident in the hospital was 111, and the average yearly cost of provisions was £21 7s. 9d. per bed occupied. The average length of stay in the hospital was a little over three weeks. The total expenditure was £7,996, and the ordinary receipts were £6,809. The administrative block has been reorganised during the year, and a new operating theatre has been opened. The former cost about £2,000 and the latter about £2,500. From the table showing the principal operations performed during 1899 we note that there were 22 amputations (7 being of the thigh) and only one death; there were 17 excisions (9 being of the knee and 4 hip), and all recovered. Of 40 cases of hernia 3 died. The honorary medical and surgical staff consists of Sir W. Acland, Sir J. Burdon-Sanderson, Drs. Tuckwell, Gray, Briscoe, Winkfield, Brooks, Collier, Ritchie, Symonds, Farmer, Whitelocke, Doyne, and Bevers. Mr. Hunt is house physician, and Mr. Harratt is house surgeon. Miss A. J. Watt is the matron.

